# Mailing Poisons

**Published:** 1916-09

**Authors:** 


### Mailing Poisons.

Just why "all kinds of poisons, and all articles and compositions containing poison" should be excluded from the mails can hardly be understood by anyone who has studied the question. Many good medicinal remedies are poisons or contain poisons. They can be sealed and mailed in such a way that they are positively harmless to those handling them, so that no danger whatever is incurred by postoffice employes and carriers. It is important often that poisons and articles containing poisons should be quickly transmitted for the treatment of diseases. The government is a public carrier, and while entitled to the protection that should be thrown about other public carriers, it can claim no more for its employes.

The National Association of Manufacturers of Medicinal Products has presented to Congress an amendment to the postal laws that will permit the mailing of poisons and drugs or articles containing poisons, when they are packed and prepared for mailing according to the directions of the postoffice department. The proposed amendment safeguards the postal employes, and provides for a much needed change that should have the quick approval of Congress.

